# Diffraction Enhanced Imaging Analysis with Pseudo-Voigt Fit Function

**DOI:** 10.3390/jimaging8080206

**Published:** 2022-07-23

**Authors:** Deepak Mani, Andreas Kupsch, Bernd R. Müller, Giovanni Bruno

**Affiliations:** 1 Bundesanstalt für Materialforschung und -Prüfung (BAM), Unter den Eichen 87, 12205 Berlin, Germany; andreas.kupsch@bam.de (A.K.); bernd.mueller@bam.de (B.R.M.); giovanni.bruno@bam.de (G.B.); 2 Non-Destructive Testing Int., Engineering Department, Dresden International University (DIU), Freiberger Str. 37, 01067 Dresden, Germany; 3 European Synchrotron Radiation Facility (ESRF), 71, Avenue des Martyrs, 38043 Grenoble, France; 4 Institute of Physics and Astronomy, University of Potsdam, Karl-Liebknecht-Str. 24–25, 14476 Potsdam, Germany

**Keywords:** diffraction enhanced imaging, analyzer-based imaging, X-ray refraction, non-destructive evaluation, Pseudo-Voigt fit function, Python

## Abstract

Diffraction enhanced imaging (DEI) is an advanced digital radiographic imaging technique employing the refraction of X-rays to contrast internal interfaces. This study aims to qualitatively and quantitatively evaluate images acquired using this technique and to assess how different fitting functions to the typical rocking curves (RCs) influence the quality of the images. RCs are obtained for every image pixel. This allows the separate determination of the absorption and the refraction properties of the material in a position-sensitive manner. Comparison of various types of fitting functions reveals that the Pseudo-Voigt (PsdV) function is best suited to fit typical RCs. A robust algorithm was developed in the Python programming language, which reliably extracts the physically meaningful information from each pixel of the image. We demonstrate the potential of the algorithm with two specimens: a silicone gel specimen that has well-defined interfaces, and an additively manufactured polycarbonate specimen.

## 1. Introduction

The use of lightweight materials for various applications has rapidly increased in recent years. To utilize these materials in practice, a comprehensive investigation is required to validate their different properties. Since the introduction of digital detector arrays for non-destructive testing, novel possibilities to analyze and characterize materials have been available for the user. Nevertheless, hard X-rays are not suitable to analyze lightweight materials due to their poor absorption contrast. As an alternative to absorption, other interactions of X-rays with matter, such as X-ray refraction (XR) [1], can be used to obtain contrast-enhanced images of internal interfaces. This allows not only characterization, but also non-destructive evaluation (damage analysis) of these materials. Generally, XR imaging is based on the assumption that when X-rays pass through a specimen with interfaces, they are mainly absorbed and refracted at the interfaces [2], with negligible diffraction or Compton scattering. Several XR imaging techniques have been developed in recent decades. They employ devices such as gratings [3,4] or crystals [5,6,7] to separate the deflected (refracted) beam portions from the primary radiation, or use large distances between the specimen and the detector [8,9,10]. One such imaging technique is the diffraction enhanced imaging technique [6] (also known as analyzer-based imaging, ABI). It is based on the concept that an analyzer crystal acts as angular filter to obtain different images corresponding to different refraction angles.

While the XR techniques listed above are mainly synchrotron based, the roots of XR imaging date back to the 1980s and 1990s [1,11] when access to synchrotron sources was rather limited. Laboratory XR imaging techniques are based on scanning the specimen through a finely collimated beam and detecting refracted beam portions. A so-called refraction value (see Section 2) has been introduced to correlate the measured (refracted) intensity with the specific surface inside the specimen (internal surface per unit volume). When transferring the concept of the refraction value to the DEI set-up, parameters of the so-called rocking curves (RCs) are needed. It turned out that fitting conventional Gaussian peak functions did not sufficiently simulate the measured RCs and sometimes even failed completely, thereby yielding wrong values of the above-mentioned parameters. In this study, we introduce a novel robust fitting routine based on Pseudo-Voigt functions. We also demonstrate how this routine allows the extraction of the RCs parameters and, therefore, of the microstructural properties of specimens.

## 2. Diffraction Enhanced Imaging

**Figure 1:**
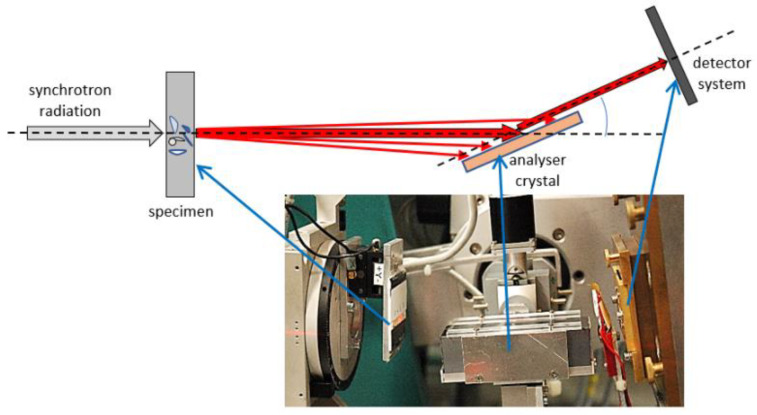
The main components of diffraction enhanced imaging technique; sketch and photograph of the actual set-up at BAM*line* (hard X-ray beamline at the electron storage ring BESSY II, at Helmholtz-Zentrum Berlin, Germany).

**Figure 2:**
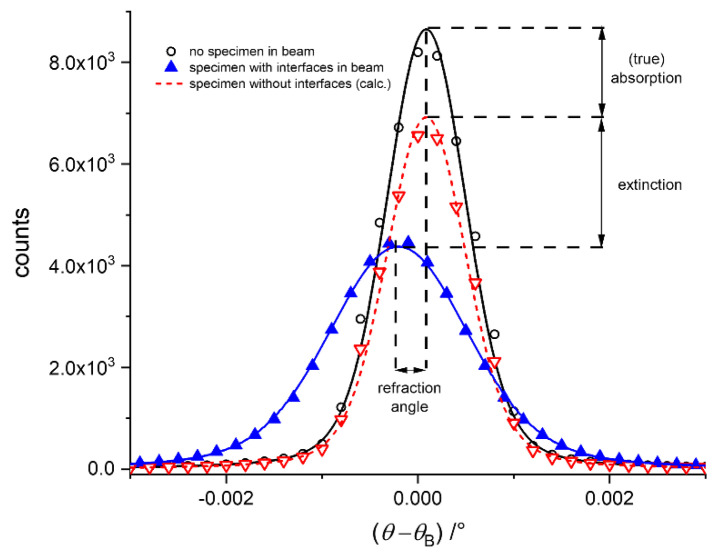
Typical RC recorded for a pixel: intensity as a function of the angular position (*θ* − *θ*_B_) of the AC. Black line: the primary beam, flat field, measured; red line: a purely absorbing specimen without any internal surfaces, calculated; blue line: an absorbing specimen containing internal surfaces, measured. The loss of (peak) intensity due to refraction can be used to quantify the scattering.

The DEI technique uses an intense and parallel monochromatic X-ray beam from a synchrotron source and an analyzer crystal (AC) positioned downstream of the specimen (see Figure 1). The AC is tilted in the (angular) vicinity of a Bragg reflection at *θ*_B_, collecting a rocking curve (RC) for each image pixel. If a (refracting) object is placed upstream of the AC, the RC is damped, broadened, and shifted. The AC separates the beam portions according to the deflection angle, *θ*’ = *θ* − *θ*_B_ (see Figure 2), by accepting only rays that meet the Bragg condition for the actual tilt angle. However, the series of images obtained using this technique need to be processed to retrieve maximum information from the RCs. The reciprocal lattice vector ***H*** of a certain AC Bragg reflection predefines the directional sensitivity of DEI experiments as follows: only features laying nearly perpendicular to the scattering plane (the plane spanned by the incident beam direction and ***H***) are detectable, i.e., not all interfaces are contrasted at once for one specimen orientation. One needs at least two perpendicular specimen orientations to detect all interfaces contained in the specimen. This, on the other hand, turns to great advantage, since it implies an orientation sensitivity of the technique: a specimen can be purposedly mounted in such a way that only interfaces of a certain orientation are contrasted.

DEI has been used to detect and quantify the microcrack density and orientation in porous ceramics [12,13] or damage after cyclic loading in all-oxide ceramic matrix composites [14]. In-situ experiments revealed the formation of cracks in fiber-reinforced plastics [15], the damage evolution in metal matrix composites under tensile load [16], or the evolution of different types of porosity in additively manufactured AlSi10Mg during heat treatment [17]. Recently, a synchrotron X-ray refraction tomography study quantified the hydrogen-assisted microcracking in duplex stainless steel [18] by 3D XR imaging.

In this study, we show that the Pseudo-Voigt function (PsdV) is suited to fit the typical RC at each pixel separately, thereby allowing the extraction of relevant microstructural features. Furthermore, the comparison of feature extraction with algorithms in ImageJ and Python elucidates the advantages of using the latter to obtain improved results with shorter computation times. Using our algorithm, we can extract and compute different parameters from the RCs; their corresponding physical information is listed in Table 1.

The RC, i.e., *I*(*θ*), measured with a specimen, corresponds to the convolution product of a reference RC, *I*_0_(*θ*), of the empty beam (the so-called flat field) and the scattering function of the specimen, *s*(*θ*):

(1) $$I\left(\theta \right)=s\left(\theta \right)⨂I_{0}\left(\theta \right),$$

where ⨂ denotes the convolution operator.

Therefore, the RC parameters of Table 1 must be determined separately for the two-image series: with and without the specimen. To extract the parameters of *s*(*θ*) (i.e., the specimen properties) we use the following equations:

(2) $$T=\int s\left(\theta \right)\;d\theta =\int I\left(\theta \right)\;d\theta \;/\int I_{0}\left(\theta \right)\;d\theta ,$$

(3) $$AT=\max\left(I\left(\theta \right)\right)/\max\left(I_{0}\left(\theta \right)\right),$$

(4) $$\Delta \theta \left(s_{\max}\right)=\theta \left(I_{\max}\right)-\theta \left(I_{0,\max}\right),$$

(5) $$w\;\left(s\left(\theta \right)\right)={\left(w^{2}\;\left(I\left(\theta \right)\right)-w^{2}\;\left(I_{0}\left(\theta \right)\right)\right)}^{1/2}$$

where Equation (2) represents the transmission *T*, Equation (3) the so-called apparent transmission *AT*, Equation (4) the refraction angle, and Equation (5) the scattering. The transmission accounts for the absorption effects only, whereas the apparent transmission accounts for absorption *and* extinction (due to scattering) effects.

In the first DEI paper [6], the authors suggest an algorithm to extract images of the “apparent absorption” and “refraction angle”. Since, from a medical point of view it is desirable to minimize the radiation dose, the authors in [6] acquired just two images (at roughly half the maximum peak intensity on each side of the RC) and used relative changes of intensity and the slope to compute the two parameters. Assuming a Gaussian RC shape, Arfelli et al. [19] introduced the G^2^DEI algorithm to additionally extract the scattering width from three images on the RC. Chen et al. [20] indicated how to retrieve different types of information from the analysis of the moments of RCs, in particular the skewness (3rd moment) and the kurtosis (4th moment), revealing the asymmetry (caused by the local curvature of interfaces) and the strength of the tails relative to the peak, respectively. Interestingly, the straightforward approach of Chen et al. does not require any fitting procedure or any assumption about the type of peak function.

In former studies, we quantified the results obtained from DEI in terms of the refraction value *C*_m_. Such quantity has been introduced by Fensch-Kleemann et al. [21] to quantify laboratory-based XR topography [11] results (i.e., 2D scans of the specimen). In this laboratory set-up, the refracted beam portions (see e.g., [22,23,24,25]) deviating from a well-collimated primary beam (collimation of the Kratky type [26]) are collected in a fixed angular range. The *C*_m_ can be calculated by normalizing the intensity of the refracted X-rays with the corresponding absorption effects. This formalism has been transferred to the quantities obtained from DEI experiments [16], assuming that *C*_m_ can be considered as an additional attenuation coefficient; see Figure 2 (for details see [16]). In terms of RC parameters, the sample transmission corresponds to the ratio of peak integrals of *I*(*θ*) and *I*_0_(*θ*) (*I*_T_ and *I*_T0_ in Equations (6) and (7)). A purely absorbing specimen would just dampen the RC without the specimen, *I*_0_(*θ*), by a constant factor, leaving the width unchanged (see Figure 2). Since refraction corresponds to a pure redistribution of beam portions (i.e., it leaves the integral unchanged), a specimen with internal interfaces causes broadening of the RC at the expense of peak height (extinction). Therefore, the refraction value represents an equivalent to the scattering as follows:

(6) $$C_{m}\cdot d=1-\frac{I_{R}}{I_{R0}}\frac{I_{T0}}{I_{T}},$$

where *d* is the specimen thickness, *I*_R_ = max(*I*(*θ*)) and *I*_R0_ = max(*I*_0_(*θ*)) are the RC peak heights with and without the specimen, respectively. In other words, *C*_m_ is the apparent transmission normalized to the true transmission. In order to eliminate the dependence on the specimen thickness, Equation (6) is normalized by *µ·d* = −ln(*I*_T_/*I*_T0_) = −ln(*T*), *µ* being the attenuation coefficient as follows:

(7) $$C_{m}/µ=\left(1-\frac{I_{R}}{I_{R0}}\frac{I_{T0}}{I_{T}}\right)/\ln\left(\frac{I_{T0}}{I_{T}}\right).$$

This approach waives one recording full RCs, when such measurements are exceedingly time consuming, e.g., in the case of X-ray refraction computed tomography (XRCT) experiments [18,27]. Instead of sampling the full RC, one can record images with the AC at *θ*_B_ (i.e., in the RC center of the flatfield, without the specimen). At this setting, all X-rays deflected by refraction events within the specimen are rejected by the analyzer crystal, causing an additional attenuation of the X-rays; assuming negligible peak shifts one could write: *I*_R_ = *I*(*θ*_B_) and *I*_R0_ = *I*_0_(*θ*_B_)). Transmission images are recorded as conventional radiographs, i.e., without the AC in the beam and with the detector lowered into the (incident) beam. This set-up yields *I*_T_ and *I*_T0_. In this study, however, *I*_R_(*θ*_C_) is the maximum of the fitted RCs (i.e., *θ*_C_ being *θ*(*I*_max_)).

## 3. Experimental Section

The DEI experiments were carried out at the beamline BAM*line*, using synchrotron radiation of the electron storage ring BESSY II, at Helmholtz-Zentrum Berlin, Germany [28,29]. Using a double crystal monochromator (Si (111)), the beam energy was set to 17.5 keV. A flat panel detector (1600 × 1200 pixels) in combination with a lens system and a 50 μm thick CWO scintillator screen provided a pixel size of 4.08 µm, capturing a field-of-view (FoV) of approximately 6.5 × 4.9 mm^2^. The incident beam was narrowed to the FoV by a slit system, to avoid detector backlighting [30,31]. The exposure time for each image of the series was 1 s. The AC was adjusted to *θ*_B_ = 6.488°. The RC was sampled in 61 steps of 2 × 10^−4^° (~3.5 µrad) symmetrical to *θ*_B_. Two such image series were recorded with and without (the so-called flat field) the specimen in the beam path. Beyond numerical normalization, the flat field images enable one to remove strong artefacts of the imaging system (composed by the monochromator, the AC, and the scintillator screen). Examples of raw images obtained from different tilt positions of the AC are shown in Figure 7 below.

In our set-up, the scattering vector pointed upwards. In the images shown below, refraction contrast was obtained only from interfaces, the normal of which has a vertical component.

In Figure 2, the changes in the RC due to a purely absorbing specimen (without any interfaces) is a pure reduction of the RC (i.e., the width remains a constant red line), while an absorbing and refracting specimen (i.e., with internal surfaces) broadens the curve (blue line). The integral of the two RCs is the same. In some cases, we additionally observe a shift of the peak position (also depicted in Figure 2). The RCs are recorded for each individual pixel of the entire image of 1600 × 1200 pixels.

The total number of RCs per image (~2 million) demands the use of an automated and robust algorithm to extract the RC parameters by using appropriate computer programming languages.

A silicone gel specimen containing air bubbles serves as a demonstration to highlight the properties that can be extracted (here, very well-defined and separated interfaces).

Additionally, an additively manufactured (AM) polycarbonate specimen [32] displays different features (i.e., elongated pores at the interface of consecutive layers).

## 4. Image Analysis Algorithm

### 4.1. Selection of Fitting Functions

**Figure 3:**
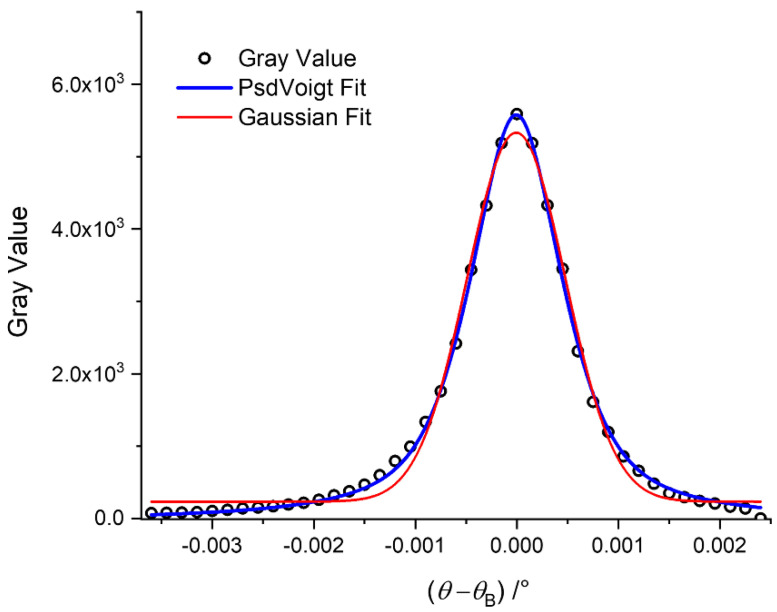
Comparison of the two fitting functions to the experimental RC of a single pixel. The gray value (intensity of the pixel at that angular position of the analyzer crystal) is plotted as a function of the number of slice (proportional to the angular position of the analyzer crystal).

As a reference, we implemented a fitting procedure based on Gaussian functions (Equation (8)), as suggested, e.g., by Arfelli et al. [19]:

(8) $$I\left(\theta \right)=I_{B}+\frac{Ae^{\frac{-4\ln\left(2\right){\left(\theta -\theta_{c}\right)}^{2}}{w^{2}}}}{w\sqrt{\pi /4\ln\left(2\right)}},$$

where *I*_B_ is the baseline level (offset), *θ*_c_ is the center, *A* is the integral, and *w* the FWHM of the RC for each pixel. The FWHM is linked to the standard deviation *σ* by the factor (8 ln(2))^½^. It should be noted that *θ*_c_ is not necessarily equal to *θ*_B_, in particular in the presence of macroscopic structures which cause a peak shift. It turned out that fitting with Gaussians could not properly reproduce the RCs at the maximum and at the tails at the same time (Figure 3).

Among different alternative peak fitting functions, such as Pearson VII or Inverse Polynomial, the Pseudo-Voigt (PsdV) fit function proved to be best suited to fit the experimental RCs and extract the targeted quantities. The Voigt function is a convolution of Gaussian and Lorentzian functions, whereas the PsdV function (Equation (9)) is a simpler weighted sum of these two functions, as a close approximation to the Voigt function.

The Pseudo-Voigt Function reads as follows:

(9) $$I\left(\theta \right)=I_{B}+A\left[m_{u}\frac{2}{\pi \;}\frac{w}{4{\left(\theta -\theta_{c}\right)}^{2}+w^{2}\;}+\left(1-m_{u}\right)\frac{\sqrt{4\ln2}}{\sqrt{\pi }\;w}\;e^{-\frac{4\ln\left(2\right)}{w^{2}}\;{\left(\theta -\theta_{c}\right)}^{2}}\right],$$

where *m*_u_ is the (linear) weighting factor of Lorentzian and Gaussian functions.

**Figure 4:**
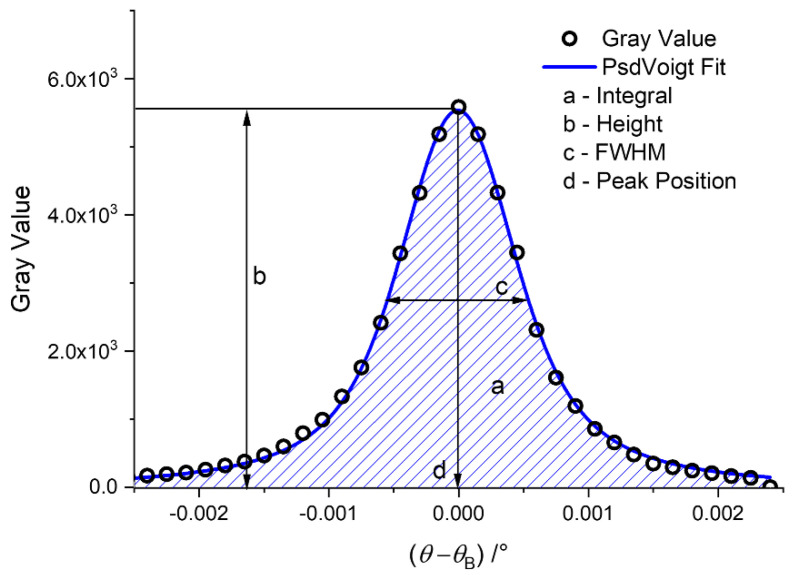
Experimental RC of a single pixel (black dots) and the PsdV function (blue line). The letters indicate the quantities to be extracted.

Only three out of five different coefficients obtained from the PsdV function proved to be useful for further evaluation, namely, *A*, *w*, and *θ*_c_ (Figure 4). They resulted in different image modalities (Table 1). The height of the RC gives the information about the reduction of intensity due to refraction, and also needs to be extracted. As the PsdV function does not contain a parameter to directly extract this value, we coded the algorithm to calculate *I*_max_ from the fitting parameters (see Section 4.3) using the following equation:

(10) $$I_{\max}=I_{B}+2A/\pi w\left(\left(1-m_{u}\right)\sqrt{\pi \;\ln2}+m_{u}\right).$$

It is obvious that the PsdV function results in much better fit of the tails and of the peak of a typical experimental RC, compared to the Gaussian function. This is supported by the calculated *R*^2^ values, serving as a figure of merit: 0.9917 for the Gaussian and 0.9988 for the PsdV.

### 4.2. ImageJ Macro Language

The PsdV fitting procedure was first implemented using ImageJ Macro to validate the accuracy of the fit, as it can be computed with simple codes. Although ImageJ [33] offers a variety of inbuilt peak fitting functions, the PsdV function had to be implemented as a user-defined plugin. By fitting and analyzing the RC of every pixel of the image and flat field stacks with the PsdV function, the extracted quantities (the parameters of the function) were then assigned as gray values to the respective pixel of the different image modalities.

The PsdV function requires initial guesses for each of its parameters. These initial guesses were tested using Origin 2019 software, such that the fitting procedure could converge with the minimum number of iterations, in the minimum time, and with optimal quality for each pixel. Changes and manipulation of these guesses had a drastic impact on the fitting of the curve. Based on numerous trials using Origin 2019, the accuracy of the initial guesses for the fitting were checked and were also validated for use in ImageJ. Since the RC height is needed for the evaluation, it was derived from a numerical fine sampling of the obtained fit function (i.e, with a step five times finer than the experimental angular steps).

The four images (see Table 1) of the specimen and the flat field were used to calculate images of: the transmission (peak integral), the apparent transmission (peak height), the refraction angle (peak position) (according to Equations (2)–(5)), the refraction value *C*_m_·*d*, the attenuation *µ*·*d*, and the specific surface *C*_m_/(*µ*·*d*), according to Equations (6) and (7). It should be noted that the negative logarithm of the apparent transmission image is named the apparent absorption in some studies (e.g., [6,19]).

**Figure 5:**
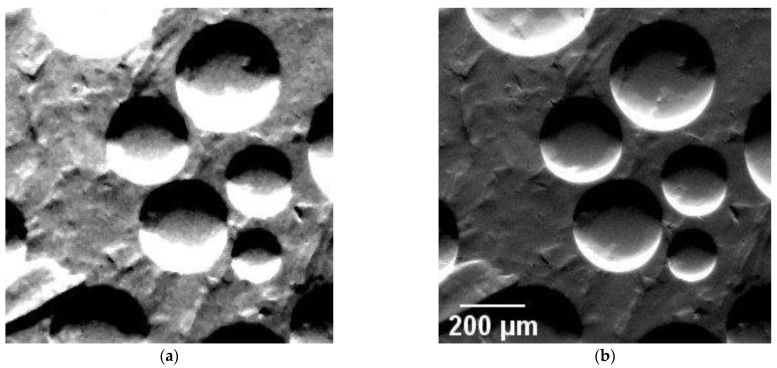
Refraction angle images of the silicone gel specimen obtained from (**a**) the Gaussian function and (**b**) the PsdV function. Note the substantial improvement in the quality of image (**b**), which was computed using the PsdV function.

Figure 5 shows refraction angle images computed using the ImageJ Macro Language, using the two types of fit functions in the case of the silicone gel specimen.

**Figure 6:**
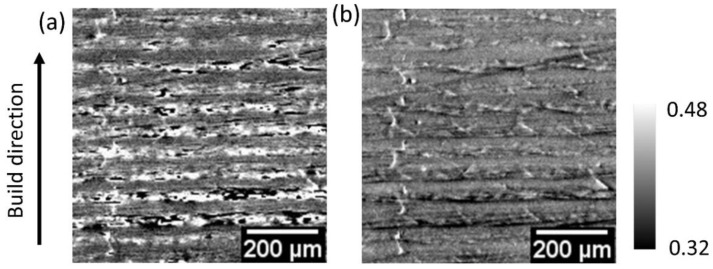
Comparison of transmission (integral) images of an additively manufactured polymer specimen obtained from fitting PsdV functions in (**a**) ImageJ and (**b**) Python. The black pixels in Figure (**a**) are due to ill-fitting of the experimental RC. Figure (**b**) demonstrates the improved quality of images with no ill-fitted pixels using the Python module.

For larger datasets (number of images in a stack), the computing time per iteration increased, and some erroneous fit results occurred. Figure 4 shows that the peak region of the experimental RC is not exactly fitted at the peak. This phenomenon occurred for the AM specimen; in that case, the different images modalities displayed some erroneous pixels (see Figure 6a).

The reason for this failure of the fitting procedure was the ad-hoc implementation of the user-defined macro.

Instead of using Equation (9) for each actual AC tilt angle, it proved advantageous to compute the fitting as a function of the slice number (i.e., the integer image number in the stack). Each image was read as the number of the slice, which is proportional to the angle of the analyzer crystal. The fitting function used this number to calculate the variables of the function, rather than reading the angle itself.

As the number of images in a stack are increased, computation times become longer. Therefore, after the accuracy of the fitting procedure was validated by means of *R*^2^ values, the task of reducing the computing time had to be addressed. In fact, even though the images evaluated with the PsdV function were of better quality as compared to the Gaussian, the use of the PsdV led to an approximately 25 times large computing time for each pixel than the Gaussian function. This factor increased even further with the image size and with the number of images in the stack in ImageJ. Table 2 lists the iteration parameters for both types of peak functions for one pixel.

The total time required to evaluate the whole specimen and the flat field stack reached 2–3 days (depending on the number of images in the stack). Table 3 lists the influence of the number of images in the stack on the time required for computation. It is to be noted that all times reported are intended as average values. Every fit of an individual RC of a pixel in a stack may require a different number of iterations. To overcome these shortcomings and optimize the time performance of the algorithm, this approach was programmed in the Python language.

### 4.3. Python Code

Python 3.0 (released in 2008) is a multi-paradigm programming language that fully supports both object-oriented programming and structured programming. The vast array of standard libraries in Python could provide a better solution to both problems: increase the quality of images and reduce the computing time. The *lmfit* module package contains fitting procedures of peak functions such as Gaussian, Lorentzian, Voigt, PsdV, etc. [34]. This Python module allowed the algorithm to yield images of superior quality in substantially reduced computing times.

The algorithm in Python was coded to follow the same procedure of creating blank images and assigning the parameters as gray values to the respective pixels of the image. The main part of the Python code is given in the Supplementary Material. Irrespective of the number of images in the stack, the internal features or the deviations of the RC from the ideal curve, optimal convergence of the fitting procedure (see Figure 6b) could be obtained in approximately 8 h. This represented a time reduction of a factor of 10, compared to ImageJ. Moreover, the image quality was significantly improved.

It is to be noted that the time required for the computation depends on the specifications of the computer. The computer used for this computation possesses an Intel(R) Core (TM) i5-8500 CPU at 3.00 GHz, 32 GB RAM and a 64-bit operating system. The Python program also allows simultaneous work with different sets of images, with each set working in dedicated consoles. This represents an enormous improvement as compared to ImageJ (even if several instances of ImageJ are opened simultaneously). Simultaneously working with different sets of image stacks is limited only by the specifications of the computer. In the case where eight different pairs of image stacks (object and flat field stack) were processed simultaneously, the fit converged to its optimum in 14 h (total for all of the eight pairs of images).

## 5. Results and Discussion

**Figure 7:**
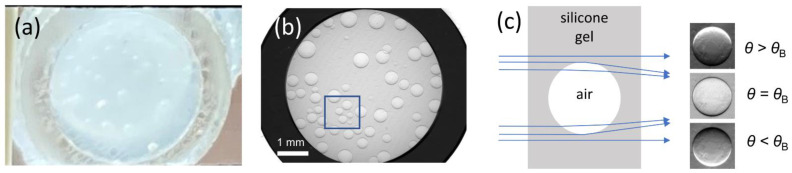
(**a**) Photograph of the silicone gel specimen with microscopic air bubbles, (**b**) raw image (corrected for the flat field) recorded in the center of the rocking curve. The marked region shows a concentration of air bubbles. (**c**) Sketch of the principal paths of X-rays traversing a spherical object, acting as an imperfect converging lens. The RC sample’s intensity accumulations from the top and bottom cap of the bubbles at different off-center AC positions and the extinction at edges in the RC center (images on the right).

**Figure 8:**
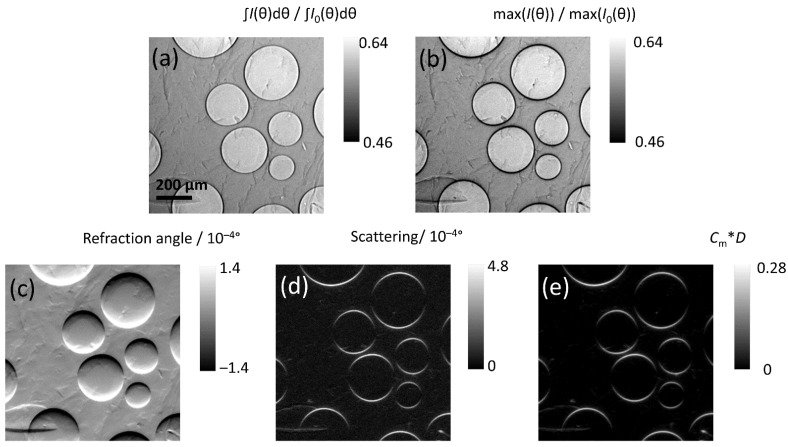
Image details computed from fitting each pixel of the specimen with the PsdV function, showing (**a**) transmission, (**b**) apparent transmission, (**c**) refraction angle (**d**) scattering (width), and (**e**) *C*_m_·*d*. The latter two quantities highlight the same features at different scales, proving the analogy derived in Section 2. Note that the scattering vector is pointing upwards, so that crescents appear above and below each bubble.

The silicone gel specimen (Figure 7a,b) with microscopic air bubbles ranging from approximately 100–400 µm in diameter, served as an example of a material meso-structure containing separated and geometrically well-defined interfaces. The images were computed with the algorithm programmed in Python, and all modalities could be extracted from the RC (Figure 8).

The principle of deflection of monochromatic X-rays at spherical shapes inside a specimen is shown in Figure 7c. The edges of an air bubble, i.e., its concave boundaries, cause the X-rays to be refracted convergently [35].

At the lower angular position, we observe the X-rays concentrated at the lower edge inside the air bubble, and at the higher angle, concentration at the upper edge. (Figure 7c). In Figure 8, a 250 × 250-pixel (1 × 1 mm^2^) detail of the full image (Figure 7b) is shown, highlighting a cluster of air bubbles. The improved quality of images obtained using the PsdV function compared to the Gaussian function is shown Figure 8 for all of the extracted features.

The *C*_m_·*d* value is computed for each pixel according to Equation (5) (basically the ratio of height and integral of the RCs normalized to the flat field). In other words, the loss of peak intensity is an indirect measure of the increase of the peak width. Thus, the image of *C*_m_·*d* and the scattering (width) carry the same information; this can be seen from a comparison of Figure 8d, e (see also Table 1).

The DEI or analyzer-based imaging technique is sensitive to the interfaces that are parallel to the plane of the analyzer crystal. (To observe the interfaces perpendicular to such plane we would have to rotate the specimen.) In the *C*_m_·*d* images, it was observed that at the top and the bottom edges of each air bubble (i.e., surface nearly parallel to the plane of the analyzer crystal) crescent shape signals appear, whereas the perpendicular interfaces (at the equator line of each air bubble) display no noticeable intensity. An advantage of the DEI technique is that the scattering vector is predefined by the analyzer crystal, therefore surfaces with different orientation (e.g., cracks and pores) can easily be distinguished and quantitatively separated. In fact, if a further measurement was performed with the orientation of the specimen changed by 90°, pores would yield the same signal (i.e., the same images as in Figure 8), while cracks would not be detected if they were visible in the previous set-up (and vice-versa, they would be detected if they were previously invisible).

## 6. Conclusions

We have shown that the physical information content of image series collected according to the DEI principle can be more precisely exploited when using PsdV functions (instead of Gaussians) to fit experimental RCs. Using the ImageJ macro code, the fitting routines were optimized with respect to accuracy, robustness, and speed. However, it turned out that, locally, some RCs could not be fitted (especially if one of the parameters of the fitting function was far from the average values in the image). Using an optimized tool (*lmfit*) in a Python programming environment, the ill-behaved RCs of such pixels could be properly fitted, and a significant reduction in the computation time (by a factor of seven compared to ImageJ) was achieved. The added advantage of working in dedicated consoles for simultaneously processing different image datasets assured the practicality and versatility of the developed Python algorithm.

Beyond the modalities (transmission, refraction angle, scattering), which have been shown to be extracted from Gaussian type RCs, we showed the analogy between scattering and the so-called refraction value (*C*_m_) images. Since the latter quantity is accessible with lower experimental effort, the use of our Python routine paves the road to an even faster access to X-ray refraction data analysis.

## Figures and Tables

**Table 1:** Extracted rocking curve parameters and their physical equivalent. FWHM is the full width at half maximum.

RC Parameter		Physical Phenomenon	Structural Information
peak integral	*∫ I*(*θ*) d*θ*	transmission	absorbing mass
peak height	*I*_max_ = max (*I*(*θ*))	apparent transmission	absorbing mass + specific surface
peak position	mode (*I*(*θ*))	refraction angle	structure gradient ^1^
peak width (FWHM)	*w* (*I*(*θ*))	scattering	specific surface

^1^ Gradient of the transmission length of the local scattering macro-structures, i.e., gradient of the objects’ phase shift.

**Table 2:** Number of cycles (iterations) needed for the best fitting for individual RC and the average time required for each cycle in ImageJ.

	Gaussian Fit	PsdV Fit
Number of iterations	106	936
Time consumption/RC	2 ms	49 ms

**Table 3:** Number of iterations needed and time consumption per RC of fitting PsdV functions in ImageJ as a function of the number of images of DEI measurements.

Number of Images/RC	Iterations (Average)	Time/RC (Average)
41	936	49 ms
61	967	60 ms
81	969	96 ms
121	1305	124 ms

## Data Availability

Any data and code used in this paper is available from the authors on reasonable request.

## Supplementary Materials

Supporting information on the main part of the Python code can be downloaded at: https://www.mdpi.com/article/10.3390/jimaging8080206/s1.
