# Supplementary material for: Diffraction Enhanced Imaging Analysis with Pseudo-Voigt Fit Function
Source: J Imaging. 2022 Jul 23;8(8):206. doi: 10.3390/jimaging8080206 (PMC9330705; doi:10.3390/jimaging8080206)
Supplement: Supplementary file 1 [file jimaging-08-00206-s001.zip › jimaging-1816673-supplementary.pdf]

Journal of Imaging

Supplementary Material

Diffraction Enhanced Imaging Analysis with Pseudo-Voigt Fit Function

The main part of the algorithm, coded in Python, is shown below. It is based on using the *lmfit* module package [34]. It shows how the experimental data of a single RC is read and implements the Pseudo-Voigt fitting model. From the fitting, the parameters are extracted, which are then assigned to that respective pixel for each image modality.

" RC_Analysis.py"
Script for Diffraction Enhanced Imaging (rocking curve) analysis with Pseudo-Voigt fit

```
…
from lmfit.models import PseudoVoigtModel
…
y = data1[ :, i, j ][ np.isfinite( data1[ :, i, j ] ) == True ]

x = np.arange( len( y ) )

mod = PseudoVoigtModel()
pars = mod.guess(y, x=x)
out = mod.fit(y, pars, x=x)

dictionary = out.params.valuesdict()

result[ 0, i, j ] = dictionary[ 'integral' ]
result[ 1, i, j ] = dictionary[ 'center' ]
result[ 2, i, j ] = dictionary[ 'fwhm' ]
result[ 3, i, j ] = dictionary[ 'height' ]
```
